# Phase II study of neoadjuvant paclitaxel and cisplatin for operable and locally advanced breast cancer: analysis of 126 patients

**DOI:** 10.1038/sj.bjc.6601616

**Published:** 2004-03-02

**Authors:** A A Ezzat, E M Ibrahim, D S Ajarim, M M Rahal, M A Raja, A M Tulbah, O A Al-Malik, M Al-Shabanah, R Sorbris

**Affiliations:** 1 Department of Oncology (MBC64), King Faisal Specialist Hospital and Research Center, PO Box 3354, Riyadh 11211, Kingdom of Saudi Arabia; 2 Department of Pathology, King Faisal Specialist Hospital and Research Center, PO Box 3354, Riyadh 11211, Kingdom of Saudi Arabia; 3 Department of Surgery, King Faisal Specialist Hospital & Research Center, PO Box 3354, Riyadh 11211, Kingdom of Saudi Arabia

**Keywords:** breast cancer, locally advanced, neoadjuvant, chemotherapy, paclitaxel, cisplatin

## Abstract

In an earlier study, we have demonstrated a high clinical and pathologic response rate of neoadjuvant paclitaxel (P) and cisplatin (C) for patients with locally advanced breast cancer (LABC). The current phase II study includes larger number of patients who had longer follow-up. A total of 126 consecutive patients with noninflammatory LABC (T2 >4 cm, T3 or T4, N0–N3, M0) were included in the study. Patients were scheduled to receive three to four cycles of the neoadjuvant PC (paclitaxel 135 mg m^−2^ and cisplatin 75 mg m^−2^ on day 1) every 21 days. Patients were then subjected to surgery and subsequently received six cycles of FAC (5-fluorouracil 500 mg m^−2^, doxorubicin 50 mg m^−2^, and cyclophosphamide 500 mg m^−2^) or four cycles of AC (doxorubicin 60 mg m^−2^ and cyclophosphamide 600 mg m^−2^); all drugs were administered intravenously on day 1 with cycles repeated every 21 days. Patients then received radiation therapy, and those with hormone receptor-positive tumours were given adjuvant tamoxifen intended for 5 years. The median age was 41 years. Clinically, 12, 52, and 37% of patients had T2 >4 cm, T3, and T4, respectively. The mean tumour size was 7 cm (95% CI, 7.3–8.5). The clinical nodal status was N0, N1, and N2–N3 in 32, 52, and 17% of patients, respectively. Disease stage at diagnosis was IIA (2%), IIB (32%), IIIA (28%), and IIIB (39%). Clinical assessment of the primary tumour and the axillary nodal status after primary chemotherapy showed that 35 patients (28%) achieved complete response (cCR), while 80 (63%) demonstrated partial response to PC. Of patients with evaluable pathologic data of the primary tumour (123 patients), complete pathologic response (pCR) was achieved in 29 patients (24%), and an additional nine (7%) only had a microinvasive disease. Moreover, 20 of the 122 patients (16%) had no residual disease in the primary tumour or in the axillary nodes. Failure to attain cCR predicted failure to achieve pCR. At a median follow-up of 37.5 months (95% CI, 31.5–43.3), 71% were alive with no recurrence, 16% were alive with evidence of disease, and 13% were dead. Of the 122 patients who had surgery, 36 (29%) developed recurrence including one of the patients who attained pCR. The median overall or disease-free survival has not been reached with a projected 5-year overall survival (OS) and disease-free survival (DFS) of 85% (±4%) and 63% (±5%), respectively. On multivariate analysis, clinical response of the primary tumour, pathological response of the primary tumour, and the pathological nodal status were identified as independent prognostic variables for DFS. No variable, however, was identified to prognosticate OS. PC was acceptably safe. Neoadjuvant PC as used in this phase II study in a multidisciplinary strategy was highly effective. Clinical and pathologic responses remain the most important variables that predict outcome.

Neoadjuvant chemotherapy has been used as the initial treatment in locally advanced breast cancer (LABC). The Milan Group, using a combination of doxorubicin and vincristine, achieved an 80% response rate, with 15% of patients attaining complete clinical response (De Lena *et al*, 1981). Other groups have achieved comparable results (Hortobagyi *et al*, 1988; Kuerer *et al*, 1999). With this approach, 5-year survival rates of approximately 40% have been reported (Valagussa *et al*, 1990), while significantly higher survival rates have been reported by others (Hortobagyi *et al*, 1988; Smith and al-Moundhri, 1998; Kuerer *et al*, 1999). Likewise, primary chemotherapy has been used successfully for large operable breast cancer in nonrandomised and randomised trials (Anderson *et al*, 1991; Smith *et al*, 1993; Scholl *et al*, 1994; Powles *et al*, 1995; Smith *et al*, 1995; Bonadonna *et al*, 1998; Fisher *et al*, 1998; Von Minckwitz *et al*, 1999). Moreover, the National Surgical Adjuvant Breast and Bowel Project (NSABB) B-18, one of the largest reported studies including 1523 patients, has clearly shown that at 9 years of follow-up, the outcome of patients who received primary chemotherapy was equal to that for patients treated with adjuvant chemotherapy (Wolmark *et al*, 2001). However, a marginally statistically significant survival advantage from neoadjuvant chemotherapy was noted for younger patients, whereas the reverse may be true for older patients.

We have demonstrated the significant efficacy of the combination of paclitaxel and cisplatin (PC) in the management of patients with metastatic breast cancer disease where an objective response of 80% was achieved (Ezzat *et al*, 1997). The results of this phase II study were in concordance with the reported efficacy of paclitaxel and cisplatin used in various settings in the management of breast cancer (Hortobagyi, 1997; Comella *et al*, 1998; Henderson *et al*, 1998; Kourousis *et al*, 1998; Perez *et al*, 1998; Volm *et al*, 1998). Furthermore, both drugs have demonstrated potency on anthracycline-resistant tumours (Lau *et al*, 1998, Ray-Coquard *et al*, 1998). The lack of apparent crossresistance between paclitaxel and cisplatin, the minimal overlapping toxicity, and the potential angiogenesis antagonistic effect of paclitaxel that appears to be independent of its antiproliferative action, make this combination very appealing for the management of LABC.

In an earlier phase II report that included 72 patients with LABC, we have demonstrated that the PC regimen was very effective and reasonably tolerant (Ezzat *et al*, 2000). Complete and partial clinical response to PC was demonstrated in 18 and 72% patients, respectively, for an overall response of 90% (95% confidence interval (CI), 79–100%). Moreover, complete pathologic response (pCR) and partial pathologic response (pPR) was achieved in 15 (22%) and 51 (75%) of patients, respectively, for an overall response of 97% (95% CI, 95–99%).

The current series update the outcome of 126 patients at a median follow-up of approximately 4 years. The primary end point was to assess the effect of that regimen on clinical and pathological response, while the secondary end point was to analyse the effect on survival, and to identify predictors of response and prognostic factors of survival.

## PATIENTS AND METHODS

Between May 1995 and December 2000, consecutive patients seen at our institution with locally advanced noninflammatory breast cancer (T2>4 cm, T3 or T4, N0–N2, M0) confirmed on fine needle aspirate (FNA) and/or tru-cut biopsy were considered eligible. Those with bilateral breast cancer, or documented evidence of metastatic disease, including supraclavicular lymph node involvement, were excluded. Also excluded were patients with a history of renal, hepatic or heart failure, history of malignancy except basal cell carcinoma of the skin or cervical carcinoma *in situ*, or history of psychiatric disorder that might interfere with the administration of protocol drugs or follow-up. The study intended to exclude patients who developed breast cancer during pregnancy or those who became pregnant during therapy; however, those events were not encountered. Patients who experienced any grade 4 toxicity, grade 3 or 4 neurotoxicity, those who requested withdrawal from the study, and those who demonstrated tumour progression after at least two cycles of the neoadjuvant regimen were removed from the study, but were included in the analysis.

Staging procedures included complete history and physical examination, laboratory studies, bilateral mammography, chest and abdominal computerised tomography, and radionuclide bone scan. Hormone receptor assay was available for most patients.

Clinical sizes of primary breast cancers and axillary nodes, if the latter were palpable, were determined separately before the administration of each cycle of PC and also before surgery. At each assessment, the product of the two greatest perpendicular diameters of the tumours in the breast and axilla was measured.

The study was approved by King Faisal Specialist Hospital and Research Center's Research Advisory and Ethics Committees, Riyadh. All patients gave a written informed consent.

### Therapy

Patients received three to four cycles of the neoadjuvant PC therapy. Paclitaxel 135 mg m^−2^ on day 1 at 0600 hours as 3-h infusion with the standard premedication (dexamethasone, anti-histamine, and H_2_ antagonist) was initiated the night before therapy. Cisplatin was administered as 75 mg m^−2^ on day 1 at 1800 hours as a 1-h infusion with standard pre- and posthydration. The timing of drug administration was chosen in conformity with the circadian rhythm concept in the first 42 patients (Harmanek and Sobin, 1987). Cycles were repeated at 3-week intervals.

Following the neoadjuvant regimen, response was assessed clinically and mammographically. Within 4 weeks after the last cycle, patients were scheduled to undergo conservative surgery or modified radical mastectomy upon the discretion of the surgeon guided by the clinical response. Axillary lymph node dissection to levels I and II was carried out aiming for excision of at least 10 lymph nodes.

Within 2–3 weeks following surgery, patients received six cycles of FAC (5-fluorouracil 500 mg m^−2^, doxorubicin 50 mg m^−2^, and cyclophosphamide 500 mg m^−2^) or four cycles of AC (doxorubicin 60 mg m^−2^ and cyclophosphamide 600 mg m^−2^); all drugs were administered intravenously on day 1 with cycles repeated at 3-week intervals. The choice between FAC 6 × *vs* AC 4 × was made based on the primary oncologist's discretion. In recent years, some patients who demonstrated pCR were given two additional cycles of PC. Any grade 1 toxicity was treated symptomatically and therapy was allowed to continue. However, grade 3 myelotoxicity required a delay in chemotherapy until bone marrow recovery, with granulocyte colony-stimulating factor (G-CSF) support on subsequent cycles.

Following adjuvant chemotherapy, patients received radiation therapy to the chest wall and axilla or to the breast and the axilla, but the supraclavicular fossa and internal mammary chains were not routinely irradiated. Adjuvant tamoxifen 20 mg orally daily intended for 5 years was given to patients shown to have oestrogen receptor (ER)- and/or progesterone receptor (PR)-positive tumour.

### Data capture

A computerised database was created to capture prospectively the following information: patients’ demographic and clinical data, laboratory and radiologic studies, disease characteristics, and neoadjuvant therapy details including clinical response and toxicity. The database also included surgery details, axillary lymph node dissection, and pathologic data including response. Also captured were postsurgery further chemotherapy, hormonal therapy, radiation therapy, recurrence, and survival status.

### Definitions

Staging was defined according to the criteria determined by the International Union Against Cancer (UICC) (Harmanek and Sobin, 1987) with group clinical and pathological staging according to the American Joint Committee on Cancer (AJCC). We adopted the criteria for LABC reported by Haagensen and Stout (1943).

After preoperative chemotherapy was administered, breast tumours were classified according to clinical response. In the absence of clinical evidence of tumour in the breast without any new lesions occurring, the response to therapy was categorised as a clinically complete response (cCR). When the reduction in the sum of the products of the largest perpendicular diameters of the breast tumour was 50% or greater without the development of newer lesions prior to surgery, the response was judged to be partial (cPR). Clinical progressive disease (cPD) was defined as any increase greater than 25% of the sum of the products of the largest perpendicular diameters of any measurable lesions, or unequivocal appearance of new lesions after a minimum of two cycles of PC. Patients whose breast tumour response did not meet the definition of cCR, cPR, or cPD were considered to have stable disease (cSD). Thus, patients with cSD could have a tumour response of less than 50% or an increase in tumour size of <25%. This classification was also used to record the response of an axillary tumour to the neoadjuvant regimen in patients who had clinically positive nodes at diagnosis. The overall clinical response of both breast and axillary tumours was determined by combining the sum of the product of the tumour measurements in both the breast and axilla. The development of a clinically suspicious ipsilateral axillary tumour during chemotherapy was considered as evidence of cPD in patients whose axilla was clinically negative at diagnosis.

A median of 15 sections of the mastectomy or lumpectomy specimen was performed; these included sections from each quadrant, from the nipple–areola complex (if appropriate), from areas of suspicious or prior tumour involvement, and from the axillary contents (a median of seven sections). Pathologic response to neoadjuvant chemotherapy was assessed according to the criteria modified from that used by Feldman *et al* (1986). Complete pathological response of the primary (pCR) was defined as the absence of any macroscopic or microscopic evidence of invasive tumour in the surgical specimen. The same definition was applied to the axillary nodes. Partial pathological response was defined as 50% or greater reduction in the sum of products of the largest perpendicular diameters of residual lesions as compared with the size of the primary tumour regardless of the nodal status; pathologic stable disease (pSD) was defined as less than 50% reduction in the sum of products of largest perpendicular diameters of the residual disease as compared with the primary tumour size regardless of the nodal status; and pathological progressive disease (pPD) was defined as any increase greater than 25% of the sum of the products of largest perpendicular diameters of residual lesions as compared with the size of primary tumour regardless of the nodal status.

Overall survival (OS) was estimated from date of diagnosis to date of last follow-up or death from any cause. Disease-free survival (DFS) was calculated from the date of definitive surgery until last contact; recurrence ‘local, regional, or distant’; occurrence of contralateral breast cancer; occurrence of second primary cancer other than in the contralateral breast; or death without evidence of breast or second primary cancer.

### Statistical methods

A two-sided Wilcoxon–Pratt test was used to compare tumour sizes before and after PC. Comparison of means and medians of continuous variables was performed using *t*-test statistics and nonparametric methods, respectively. Patient- and disease-related variables were first examined for their relation to response, while variables with a *P*-value of <0.10 were included in a stepwise multivariate logistic regression analysis to identify the independent predictors for pCR response (Cox and Snell, 1989). Age was entered both as continuous and as dichotomous values by using different cutoffs. The goodness-of-fit *χ*^*2*^, the Hosmer goodness-of-fit, and the Brown goodness-of-fit tests were used to test the hypotheses that the model at each step fits the data, that the predicted values fit the data, and that the logistic model is the best fitting model, respectively (Prentice, 1976; Hosmer and Lemeshow, 1980). Kappa's test of reliability was used to compare the agreement between clinical and pathologic response (Fleiss, 1981). Survival was estimated applying the method of Kaplan and Meier (1958), while the statistical procedure of Brookmeyer–Crowley was used to estimate the 95% confidence interval (CI) of median survival (Brookmeyer and Crowley, 1982). The log-rank test was used to assess the significance of unadjusted differences in survival (Mantel, 1966).

Exploring variables for their independent prognostic effect on survival was carried out using the multivariate stepwise Cox's proportional regression hazard model (Cox and Oakes, 1997). The relative importance of prognostic factors was measured by the chi-square values, based on the Wald test of the coefficient associated with each prognostic factor in the Cox model (Marubini and Valsecchi, 1995). Factors with larger chi-square values were more significant in each model. The chi-square value in the ranking of prognostic factors was used because its interpretation is unrelated to the coding of the covariate. The interpretation of the hazard ratio depends on the units or coding of the covariate.

The predictor with the highest level of statistical significance was used to introduce the model; other variables were then evaluated for further predictive information and added in turn, beginning with the variables with the highest level of statistical significance (i.e. the lowest *P*-values) and continuing until the *P*-value for the variable added exceeded 0.05. Continuous prognostic variables were also considered for inclusion in the model as dichotomous variables using various cutoff points only if they attained a *P*-value of ⩽0.1 in the univariate analysis. On the other hand, discrete variables with more than two categories were analysed by means of categories or indicator variables. Cases with unknown factors were excluded in the initial Cox regression analysis. If a factor did not predict for the event of interest, the cases with unknown values for that factor were again added, and the Cox regression was repeated with that particular factor dropped. Interactions between variables were not explored. In the derived model, plotting the log-minus-log survival function tested the proportionality assumption (Kalbfleisch and Prentice, 1980), and the goodness of fit was judged by plotting the cumulative baseline hazard function for residuals (Kay, 1977). We also compared the survival functions for variables after stratifying for baseline differences in additional variables (Kalbfleisch and Prentice, 1980). All reported *P*-values were two-tailed. We performed all data analyses using SAS System for Windows, release 8.02 and SPSS release 10.0.1 for Windows.

## RESULTS

According to inclusion criteria, 126 patients were accrued and all were evaluable for response and toxicity analysis. Diagnosis was made by core biopsy or FNA in 89 (71%) and 37 (29%) of patients, respectively. Their median age was 41 years (95% CI, 40.1–43.7). Patient and disease characteristics are depicted in Table 1

**Table 1:** Patient and disease characteristics

**Variables**	**No. (%)**
*Year of diagnosis*	
1995–1997	43 (34)
1998–2000	83 (76)

*Menopausal status*	
Premenopausal	100 (79)
Postmenopausal	26 (21)

*Age (years)*	
<50	103 (82)
⩾50	23 (18)

*Primary tumour*	
T2	15 (12)
T3	64 (51)
T4	47 (37)

*Clinical nodal status*	
N0	40 (32)
N1	65 (52)
N2–N3	21 (17)

*Clinical nodal size*	
Negative	40 (32)
<2.5 cm	60 (48)
More than 2.5 cm	26 (20)

*Stage*	
IIA	2 (2)
IIB	40 (32)
IIIA	35 (28)
IIIB	49 (39)

*Grade*	
G1	4 (3)
G2	55 (44)
G3	51 (40)
Unknown	16 (13)

*Vascular invasion*	
Negative	83 (66)
Positive	24 (19)
Unknown	19 (15)

*Oestrogen receptor*	
Positive	53 (42)
Negative	43 (34)
Unknown	30 (24)
*Progesterone receptor*	
Positive	47 (37)
Negative	49 (39)
Unknown	30 (24)

. Most patients were premenopausal (79%) and approximately more than two-thirds (67%) had stage IIIA or IIIB.

### Neoadjuvant treatment

The median number of cycles of neoadjuvant chemotherapy was 3 (range, 5). Five patients received two additional cycles of doxorubicin and cyclophosphamide (AC) preoperatively after inadequate response to TP.

### Clinical response

The mean primary baseline tumour size was 7 cm (95% CI, 7.3–8.5), while the mean size following chemotherapy was 2.2 cm (95% CI, 2.5–3.8). This difference was highly significant (*P*<0.0001). Clinical assessment of the primary tumour and the axillary nodal status after primary chemotherapy showed that 35 patients (28%) achieved cCR, while 80 (63%) demonstrated cPR to the primary chemotherapy. The remaining 11 patients (9%) had cSD (nine patients) and cPD (two patients). Of the five patients who achieved inadequate response to initial TP, four demonstrated cPR after the addition of AC.

### Surgery

Modified radical mastectomy was performed in 86 patients (68%), while 36 (29%) had breast-conserving surgery. One patient had simple mastectomy and, in three patients, surgery was not performed due to disease progression and/or patient refusal. Axillary nodal dissection was performed in all except four patients. Of all patients with residual primary tumour undergoing surgery, resection margins were close or focally involved in 14 patients.

### Pathological response

Pathological status of the primary tumour was known in 123 patients, while the axillary nodal status was pathologically known in 122 patients. The median number of dissected and positive axillary lymph nodes was 14 (range, 40) and 1 (range, 19), respectively. Table 2

**Table 2:** Pathological data of the 123 patients who underwent surgery

	**No. of patients: pathological nodal status**				
**Pathology of the primary tumour**	**N0**	**N1–3**	**N4–9**	**⩾N10**	**Unknown**
T0	20	8	1		
T1A	5	4			
T1B	3	4	2	2	
T1C	6	7	4	3	
T2	6	12	15	9	
T3	2	2	5	2	1

Total	42	37	27	16	1

shows that of the 123 patients, 29 patients (24%) had no invasive tumour (eight had noninvasive intraductal carcinoma *in situ*). Additionally, in nine patients (7%) only a microinvasive cancer was found and of these, five had negative axillary nodes. The table also shows that among those who had undergone surgery and had their axillary status known, the complete pathological response, where there were no invasive primary tumour and no axillary involvement, was 16% (20 of 122 patients). Additionally, five patients had negative axillary nodes and only a microinvasive cancer. The table also shows that patients with a pCR of the primary tumour were more likely to have a negative axillary lymph node status (20 of 29; 69% *vs* 22 of 93; 24%, *P*<0.0001).

Of the 37 patients who were initially diagnosed with FNA only, only two had no invasive primary tumour and no axillary involvement; however, during primary chemotherapy one demonstrated cCR and another cPR. The remaining patients were found to have either residual invasive disease (nine patients), axillary lymph node involvement (13 patients), or residual invasive tumour and axillary disease (13 patients).

Analysis of the clinical and pathological response according to the tumour receptor status was also carried out. Of the 96 patients with known ER, cCR was achieved in 10/51 (20%) ER− patients *vs* 17/45 (38%) in ER+ patients (*P*=0.048). Similarly, pCR was attained in 1/51 (2%) and in 13/45 (29%), respectively (*P*<0.0001). On the other hand, while PR status was not related to cCR, it was significantly associated with pCR (1/46 (2%) in PR− patients *vs* 13/50 (26%) in PR+ patients, *P*=0.0003). On the other hand, the stepwise multivariate logistic regression showed that failing to attain cCR was the only independent factor that predicted no pCR (odds ratio=3.02; 95% CI 1.65–5.52; *P*-value <0.0001).

Agreement between cCR and pCR of the primary tumour was evaluated. It was shown that concordance was demonstrated in 90 of the 123 patients (73%) with known pathologic data of the primary tumour. The *κ*-test was 0.313, which only suggests a moderate agreement between clinical and pathologic (Brookmeyer and Crowley, 1982).

### Adjuvant therapy

Following surgery, adjuvant chemotherapy was offered to 117 patients. In all, 60 patients received FAC, 50 received AC, and seven received TP. The median number of cycles given was 4. Following adjuvant chemotherapy, radiotherapy was derived to 120 patients (95%) and adjuvant tamoxifen intended for 5 years was offered to 65 patients (52%) following radiotherapy.

### Overall survival

**Figure 1:**
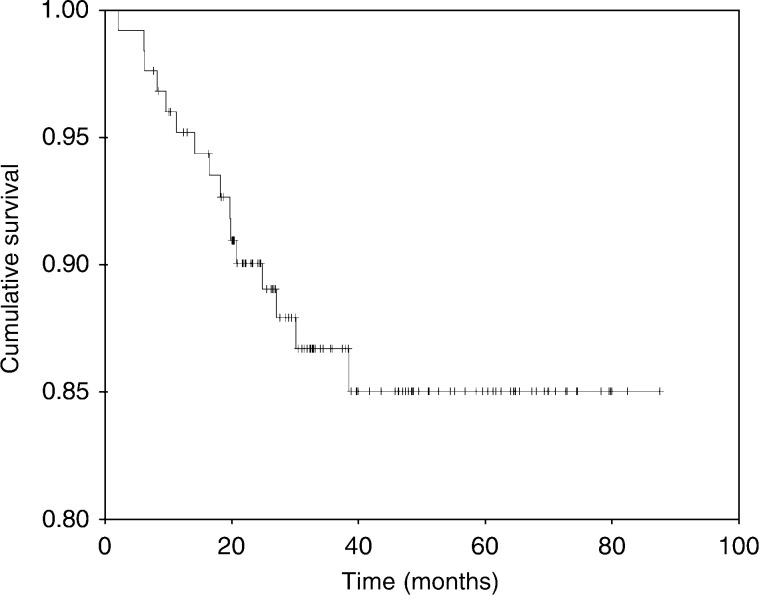
Overall survival.

At a median follow-up of 37.5 months (95% CI, 31.5–43.3), 90 patients (71%) were alive and disease-free, 20 (16%) were alive with disease, and the remaining 16 (13%) were dead. The median OS was not reached; however, the projected 5-year OS (+SD) was 85+4% (Figure 1

).

### Relapse and DFS

**Figure 2:**
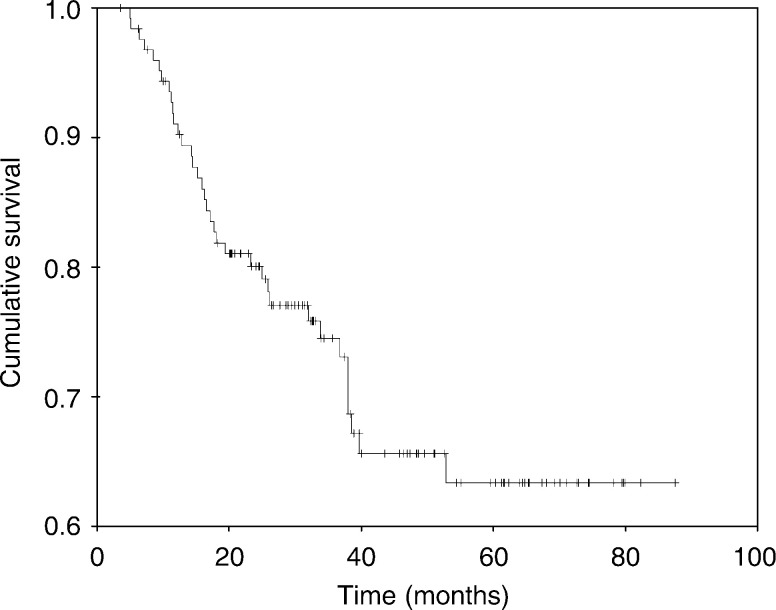
Disease-free survival.

Of all 123 patients who had surgery, 35 (29%) experienced relapse. Distant relapse only or locoregional relapse only was demonstrated in 19 (15%), and six (5%) patients, respectively. Ten patients (8%) had distant relapse concurrently with local or regional relapse. Two events of contralateral breast recurrence were experienced, one in association with local and distant relapse and the other as a lone event. Of patients with close or focally involved margins (15 patients), six did not demonstrate relapse, while four, three, and two showed local or locoregional relapse, locoregional and distant relapse, and distant relapse only, respectively. The median DFS was not reached; nevertheless, the projected 5-year DFS (+SD) was 63+5% (Figure 2

).

### Univariate and multivariate analyses of prognostic factors of OS and DFS

Table 3

**Table 3:** Univariate analyses of the effect of variables on overall survival (OS) and disease-free survival (DFS)

	**OS**		**DFS**	
**Variable**	**No. (% survival)**	***P*-value**	**% DFS**	***P*-value**
*Age (years)*		0.36		0.22
<50	103 (88)		73	
>50	23 (83)		55	

*Menopausal status*		0.52		0.004
Premenopausal	100 (88)			
Postmenopausal	26 (85)			

*Clinical primary tumour (baseline)*		0.01		0.14
T2	15 (93)		80	
T3	64 (94)		80	
T4	47 (77)		57	

*Clinical nodal status (baseline)*		0.01		0.02
N0	40 (90)		78	
N1	65 (92)		72	
N2+N3	21 (67)		57	

*Clinical stage (baseline)*		0.023		0.41
II	42 (92)		79	
IIIA	35 (94)		80	
IIIB	49 (78)		59	

*Tumour grade (known in 110 patients)*		0.43		0.20
G1+G2	59 (88)		73	
G3	51 (82)		63	

*Oestrogen receptor*		0.35		0.84
Positive	53 (87)		64	
Negative	43 (93)		81	
Unknown	30 (80)		70	

*Progesterone receptor*		0.53		0.07
Positive	47 (89)		74	
Negative	49 (90)		69	
Unknown	30 (80)		70	

*Diagnosis to primary chemotherapy (days)*		0.57		0.04
<21	94 (86)		76	
>21	32 (90)		59	

*Clinical primary tumour (postneoadjuvant chemotherapy)*		0.004		0.64
T0	37 (84)		70	
T1	25 (100)		80	
T2	40 (93)		77	
T3+T4	24 (71)		54	

*Clinical nodal status (postadjuvant chemotherapy)*		0.28		0.41
N0	97 (89)		72	
N1+N2	29 (83)		69	

*Clinical response*		0.72		
cCR	35 (89)		77	
<cCR	91 (87)		69	

*Tumour pathologic response (123 patients)*		0.026		0.0002
T0	29 (97)		93	
T1	40 (93)		80	
T2	43 (84)		56	
T3	11 (73)		55	

*Primary tumour pathologic response (123 patients)*		0.09		0.002
pCR	29 (97)		93	
<pCR	94 (86)		66	

*Pathological nodal status (122 patients)*		0.08		0.0001
Negative	42 (95)		93	
Positive	80 (86)		62	

*Pathological nodal status (122 patients)*		0.047		<0.0001
Negative	42 (95)		93	
1–3 positive nodes	37 (92)		73	
4–9 positive nodes	27 (85)		59	
More than 9 positive nodes	16 (75)		44	

*Pathologic stage (postneoadjuvant chemotherapy)*		0.017		0.0006
T0N0	20 (95)		95	
I	13 (100)		100	
II (A+B)	81 (86)		64	
III (A+B)	12 (67)		50	

*Adjuvant radiotherapy*		0.029		0.01
Yes	120 (88)		73	
None	6 (67)		33	

*Adjuvant tamoxifen (receptor-positive patients only, 57)*		0.46		0.51
Yes (46 patients)	42 (91)		70	
No (11 patients)	9 (82)		63	

shows the univariate analysis of prognostic factors of OS and DFS. For OS, the following variables were statistically significant: clinical primary tumour stage, nodal status at baseline, clinical baseline stage, postneoadjuvant chemotherapy clinical primary tumour stage, pathological primary tumour, pathological nodal status, pathological stage, and adjuvant radiotherapy. On the other hand, the variables that were significant prognostically for DFS were: clinical primary tumour stage, clinical baseline stage, postneoadjuvant chemotherapy clinical primary tumour stage, pathological primary tumour, pathological nodal status, pathological stage, and adjuvant radiotherapy.

The multivariate stepwise Cox's proportional regression hazard model failed to identify independent prognostic factors for OS. On the other hand, clinical response of the primary tumour (T0 *vs* >T0; odd ratio=0.60 (95% CI 0.39–0.94)), pathological response of the primary tumour (T0 *vs* >T0; odd ratio=0.39 (95% CI 0.25–059)), and the pathological nodal status (negative *vs* positive; odd ratio=0.18 (95% CI 0.05–0.60) were identified as independent prognostic variables for DFS.

### Toxicity

The combination of PC was well tolerated with only rare events of grade III or IV toxicity. Hypersensitivity reaction to paclitaxel was reported in three patients and was severe in one with hypotension, bronchospasm, and seizure. After recovery, paclitaxel was not resumed. Myelosuppression was minimal; grade III neutropenia and anaemia developed in 11 and 2% with no grade III thrombocytopenia. No infection was observed concomitantly to grade III neutropenia. No treatment-related death occurred during chemotherapy. Nausea and vomiting were reported in 8%. Alopecia was universal. Other grade I–II toxicities included reversible renal impairment, electrolytes disturbance, ototoxicity, disorders, peripheral neuropathy, and extravasations. Dizziness, fatigue, and myalgia were commonly reported. Three patients had their third cycle of chemotherapy delayed by at least 1 week and all cycles but one were given at full dose.

## DISCUSSION

Patients with LABC do very poorly when treated by locoregional therapy alone, with less than 10–20% of these patients surviving 5 years. Such therapy favourably affects locoregional control, but most relapses are due to the development of distant metastases (Zucali *et al*, 1976; Bruckman *et al*, 1979). Recently, however, neoadjuvant chemotherapy regimens have been shown to have a favourable influence on the outcome of patients with LABC (De Lena *et al*, 1981; Hortobagyi *et al*, 1988; Valagussa *et al*, 1990; Anderson *et al*, 1991; Smith *et al*, 1993; Scholl *et al*, 1994; Powles *et al*, 1995; Smith *et al*, 1995; Bonadonna *et al*, 1998; Fisher *et al*, 1998; Kuerer *et al*, 1999; Von Minckwitz *et al*, 1999; Wolmark *et al*, 2001).

Complete and partial clinical response to PC was demonstrated in 28 and 63% of patients, respectively. Moreover, the primary tumour showed no invasive component in 24% of patients and only microinvasive disease in an additional 7%; besides, among patients who had surgery and axillary dissection, 16% patients had no microscopic evidence of invasive cancer in their breast and axillary specimens. In the latter group, only one patient subsequently developed recurrence. While it may be possible that the two patients who had no pathological evidence of disease post neoadjuvant chemotherapy and were originally diagnosed using FNA only might have had *in situ* disease, it is rather unlikely considering the demonstrated clinical response during induction therapy.

The 16% rate of complete eradication of invasive disease is comparable to the 12% rate achieved using four cycles of FAC preoperatively in 372 patients in the MD Anderson study (Kuerer *et al*, 1999). Moreover, similar to the findings in the latter series, our data also showed that a pathologic complete primary tumour response was predictive of a complete axillary lymph node response (*P*<0.0001).

Considering that adverse prognostic features prevailed in patients in the current series, which includes a median tumour size of 7 cm, the current data provide further support to our earlier conclusion about the efficacy of primary PC for patients with LABC (Ezzat *et al*, 2000). At a median follow-up of 37 months, 71% were alive and disease-free, 16% were alive with disease, and the remaining 13% were dead. The projected 5-year OS and DFS was 85 and 63%, respectively. These survival data are comparable to those results reported in other series that included patients with more favourable prognostic characteristics (Bonadonna *et al*, 1998; Fisher *et al*, 1998; Kuerer *et al*, 1999; Wolmark *et al*, 2001). As a large number of patients received adjuvant chemotherapy (92%), radiation therapy (95%), and tamoxifen (52%), the independent additional contributions of these modalities to the obtained favourable results could not be ascertained.

While ER- or PR-disease was shown to be associated with higher clinical and/or pathological response, failure to attain cCR was the only variable identified that predicts failure to achieve pCR. Nonetheless, the overall agreement between cCR and pCR was only moderate. In contrast, in a recently published series from MD Anderson, negative ER status and higher nuclear grade were independently associated with pCR (Kuerer *et al*, 1999).

While no variable could be identified to prognosticate OS independently, clinical response of the primary tumour, pathological response of the primary tumour, and the pathological nodal status were identified as independent prognostic variables for DFS. In the NSABB B-18 study, the 9-year follow-up demonstrated significant associations between clinical response and achieving pCR and OS, DFS, and relapse-free survival (Wolmark *et al*, 2001). Also shown were the associations between pathologic lymph node status and survival. In the latter study, these significant associations persisted after adjustment for clinical tumour size at randomisation, clinical lymph node status, and age.

In conclusion, neoadjuvant PC as used in this phase II study in a multidisciplinary strategy was highly effective with an acceptable safety profile. Clinical and pathologic responses remain the most important variables that predict outcome.
